# Network analysis of occupational stress and job satisfaction among radiologists

**DOI:** 10.3389/fpubh.2024.1411688

**Published:** 2024-06-17

**Authors:** Juan Ji, Bosheng He, Shenchu Gong, Meihong Sheng, Xiwu Ruan

**Affiliations:** Department of Radiology, The First People's Hospital of Nantong, The Second Affiliated Hospital of Nantong University, Nantong, Jiangsu, China

**Keywords:** occupational stress, job satisfaction, radiologists, network analysis, intricate relationships

## Abstract

**Background:**

Occupational stress and job satisfaction significantly impact the well-being and performance of healthcare professionals, including radiologists. Understanding the complex interplay between these factors through network analysis can provide valuable insights into intervention strategies to enhance workplace satisfaction and productivity.

**Method:**

In this study, a convenience sampling method was used to recruit 312 radiologists for participation. Data on socio-demographic characteristics, job satisfaction measured by the Minnesota job satisfaction questionnaire revised short version (MJSQ-RSV), and occupational stress assessed using the occupational stress scale. Network analysis was employed to analyze the data in this study.

**Results:**

The network analysis revealed intricate patterns of associations between occupational stress and job satisfaction symptoms among radiologists. Organizational management and occupational interests emerged as crucial nodes in the network, indicating strong relationships within these domains. Additionally, intrinsic satisfaction was identified as a central symptom with high connectivity in the network structure. The stability analysis demonstrated robustness in the network edges and centrality metrics, supporting the reliability of the findings.

**Conclusion:**

This study sheds light on the complex relationships between occupational stress and job satisfaction in radiologists, offering valuable insights for targeted interventions and support strategies to promote well-being and job satisfaction in healthcare settings.

## Introduction

As a specialized cohort within the medical field, radiologists encounter distinctive occupational health hazards in the unique work setting of radiology. Their responsibilities entail prolonged exposure to computer terminals for image processing and analysis, as well as extensive involvement in ultrasound examinations, which necessitate prolonged and repetitive utilization of a singular upper limb, potentially leading to the development of “Transducer User Syndrome” (1). Furthermore, interventional radiologists operating in angiography suites face specific musculoskeletal challenges (2). Recent years have witnessed significant transformations in radiology, characterized by heightened case complexities and rapid technological advancements that impose greater demands on radiologists. The formulation of treatment plans has become increasingly intricate and time-consuming, while augmented administrative and documentation requirements contribute additional stressors (3). Within this context, radiologists contend with stringent turnaround expectations and enduring work pressures, predisposing them to an increased prevalence of repetitive musculoskeletal stress injuries, persistent ocular fatigue, and physical ailments, alongside psychological well-being issues including occupational stress (4–6).

Occupational stress refers to the reaction experienced by employees in specific professions when facing job demands that exceed their physical and environmental adaptability (7, 8). This stress not only stems from the characteristics of the job itself and environmental requirements but also closely correlates with multiple factors such as organizational structure, atmosphere, information flow, role clarity, workplace relationships, perception of career development, as well as external commitments and responsibilities (9). It is worth noting that the harmful effects of occupational stress cannot be ignored. It often accompanies significant psychological and physiological distress, and may even lead to burnout, posing a serious threat to the overall well-being of employees (8). Furthermore, prolonged occupational stress can also result in employee turnover, thereby impacting the stability and operational efficiency of organizations (9). Therefore, conducting in-depth research on occupational stress and seeking effective coping strategies is of paramount importance for safeguarding employee health and enhancing organizational performance.

Job satisfaction is defined as the cognitive aspect of happiness experienced by employees in their work. According to the Job-Demands Resources (JD-R) model (10), the level of job satisfaction is determined by the available resources (11). For radiologic technologists, their job satisfaction may stem from their perception of the job’s societal importance, including their perception of the work environment, occupational prestige, job achievements, and self-realization potential. Previous studies have shown that employees who are satisfied with their work tend to perform their duties more efficiently (12, 13). Furthermore, the JD-R model suggests that the level of job satisfaction is highly correlated with the level of occupational stress, a viewpoint supported by previous research (14).

In previous studies, the exploration of the relationship between occupational stress and job satisfaction often focused on causal analysis, such as investigating through statistical methods like linear regression or logistic regression (11, 13, 15–17). However, in reality, the relationship between occupational stress and job satisfaction is not unidirectional but rather mutually influential. Occupational stress may have a negative impact on job satisfaction, while an increase in job satisfaction can help alleviate occupational stress. To comprehensively reveal this complex interactive mechanism, this study employed the latest statistical method—network analysis (18). Network analysis is a data-driven research approach that does not rely on preconceived assumptions about causal relationships between variables (19, 20). Instead, it showcases the relationships between variables by generating a network structure with spatial order. In the network, key variables occupy central positions, while variables with fewer connections are located at the network periphery. Through network analysis, we can gain insights into the degree of connectivity among variables within the same structure and how interactions and enhancements occur between different structures.

In this study, we collected multiple indicators influencing occupational stress and job satisfaction among radiologic technologists and applied network analysis to process these data. Through this approach, we aim to gain a deeper understanding of the complex interplay between occupational stress and job satisfaction, thereby providing more targeted recommendations for enhancing the work environment and occupational health of radiologic technologists.

## Method

### Participant and procedure

The data utilized in this study were gathered from a cross-sectional survey conducted in Nantong City, Jiangsu Province, between October 28 and November 7, 2022. Participants were sourced from the Nantong Imaging Professional Physicians Association. Imaging physicians meeting the following inclusion criteria were included: (1) membership in the Nantong Imaging Professional Physicians Association, (2) ability to comprehend the questionnaire’s content, (3) willingness to participate and provide informed consent, (4) absence of any diagnosed mental illnesses, and (5) current employment in the hospital’s imaging department. The study protocol underwent thorough review and approval by the Ethics Review Committee of Nantong People’s Hospital and obtained further endorsement from the Ethics Committee of Nantong People’s Hospital in China.

Participant recruitment employed a convenience sampling method, with individual information collected through a self-designed questionnaire administered via wenjuanxing software, China’s largest online survey platform. Prior to the survey, researchers underwent comprehensive training and provided a detailed explanation of the study’s objectives. The questionnaire link was distributed through the Nantong Imaging Professional Physicians Association, highlighting the importance of privacy and independence in questionnaire completion. Participants were assured of support and the right to withdraw from the study. Out of the 330 members invited, only 312 successfully completed the questionnaire, resulting in a response rate of 94.5%.

### Measurement

#### Socio-demographic variables

The study assessed socio-demographic characteristics such as age, sex, education level, marital status, annual income, and years of work experience.

#### Job satisfaction

The Minnesota Job Satisfaction Questionnaire Revised Short Version (MJSQ-RSV) to evaluate job satisfaction among participants (21). This questionnaire comprises 20 items, with 12 items focusing on intrinsic satisfaction and 8 items on extrinsic satisfaction. Respondents rated each item on a 5-point Likert scale ranging from 1 (strongly unsatisfied) to 5 (strongly satisfied). Intrinsic satisfaction measures one’s content-related job satisfaction, while extrinsic satisfaction assesses satisfaction with job rewards, promotions, and leadership style. Higher ratings indicate higher job satisfaction levels. The MJSQ-RSV scale has shown strong reliability and validity when used in the Chinese population (15, 22, 23). In this study, the Cronbach’s α was 0.95.

#### Occupational stress

The Occupational Stress Scale, developed by Chen (24), consists of 38 items divided into seven dimensions: Organizational Management (8 items), Occupational Interests (8 items), Workload (6 items), Career Development (7 items), Interpersonal Relationships (3 items), External Environment (3 items), and Doctor-Patient Relations (3 items). Participants rate each item on a scale from “Strongly Disagree” to “Strongly Agree,” with scores ranging from 1 to 4 points. Higher scores reflect increased levels of stress. In this study, the Cronbach’s α was 0.90.

### Statistical analysis

In the research, socio-demographic features were presented as Mean ± SD for continuous parameters and N (%) for categorical variables.

#### Network analysis

We used a Graphical Gaussian Model (GGM) to estimate the network, where edges denote conditional independence relationships among nodes (25). These edges can be interpreted as partial correlations, illustrating the relationship between two nodes while accounting for all other network connections. GGMs involve estimating numerous parameters (e.g., 16 nodes necessitate 136 parameter estimations: 16 threshold parameters and 120 pairwise association parameters) that may introduce false positives. To address this, GGMs are commonly regularized using graphical lasso (glasso) (26), a technique that shrinks edges and removes small ones to create a sparse, explanatory network with minimal edges. We estimated GGMs using glasso regularization along with extended Bayesian Information Criterion (EBIC) model selection for our analysis (27). Initially, 100 diverse network models with varying levels of sparsity were evaluated. The model with the lowest EBIC, determined by a specific γ hyperparameter value balancing false positives and true negatives, was selected. We set the initial γ value to 0.5 following recommendations. For visual representation, edge thickness signifies the strength of association. Node positioning relied on the Fruchterman-Reingold algorithm, placing nodes with stronger average associations closer to the graph center. Various metrics of node centrality were computed to determine the key symptoms within the network structure. Strength, which represents the total sum of edge weights linked to a node, along with closeness, indicating the average distance from a node to all other nodes in the network, and betweenness, quantifying the frequency of a node lying on the shortest path between two other nodes, were determined for each node.

To assess the precision and consistency of the combined estimated network, accuracy and stability were specifically examined. Accuracy was gauged by evaluating the confidence intervals (CIs) through bootstrapping (1,000 iterations) to determine the accuracy of the edge weights, where smaller CIs indicate enhanced precision. Stability of the node centrality and bridge centrality indices was investigated via case-dropping subset bootstrap methodology for the combined estimated network (28). This method involves evaluating the correlation between the original centrality indices and those obtained from reduced subsets with up to 75% of participants removed. Correlation stability coefficients (CS-coefficients) were computed to quantify how many cases could be dropped while still maintaining a correlation of 0.70 or higher with the original centrality indices, providing 95% confidence. Ideally, a CS-coefficient exceeding 0.25 indicates stability, with values surpassing 0.50 preferred (28).

We investigated two network characteristics that might vary between admission and discharge: the global network strength (referring to alterations in the total sum of all edges between admission and discharge) and network structure (such as shifts where highly connected nodes at admission become less connected at discharge, and vice versa, indicating significant structural changes). To assess changes in global network strength, we employed a permutation test known as the Network Comparison Test (NCT) (29).

We used R, version 4.3.1 to perform the network analysis. Visualization and computation of networks were conducted utilizing the R package qgraph. Additionally, network stability was assessed using bootnet package, and NetworkComparisonTest package was utilized to test network replicability in split samples as well as in men and women.

## Result

### Participant characteristics

A total of 312 radiologists were included in the statistical analysis. Table 1 presents the baseline socio-demographic characteristics of the participants. The mean age was 38.33 ± 10.11 years, with the majority falling within the 44–49 age group, accounting for 31.7% of the cohort. There were 172 male participants, representing 55.1% of the sample. Among them, 39 individuals (12.5%) held a bachelor’s degree or higher. A significant portion of the participants were married, with 239 individuals (76.6%) reflecting this marital status. Regarding annual income, most participants reported earnings in the range of 100,001 to 150,000 RMB, comprising 41.0% of the sample with 128 individuals falling into this category. In terms of work experience, the majority had practiced for 20–29 years, constituting 24.4% of the respondents.

**Table 1:** Socio-demographic characteristics of participants (*N* = 312).

Characteristic	Number	Percent (%)
Age (years)	38.33 ± 10.11	
20–29	77	24.7
30–39	89	28.5
40–49	99	31.7
50 ~	47	15.1
Sex		
Women	140	44.9
Men	172	55.1
Education level		
High school or lower	273	87.5
Bachelor degree or above	39	12.5
Marital status		
Unmarried	67	21.5
Married	239	76.6
Divorce/others	6	1.9
Income (year)		
10w or lower	71	22.8
10.1w ~	128	41.0
15.1w ~	68	21.8
20.1w or higher	45	14.4
Experience (years)		
0 ~	59	18.9
5 ~	58	18.6
10 ~	72	23.1
20 ~	76	24.4
30 ~	47	15.1

### Network analysis

**Figure 1:**
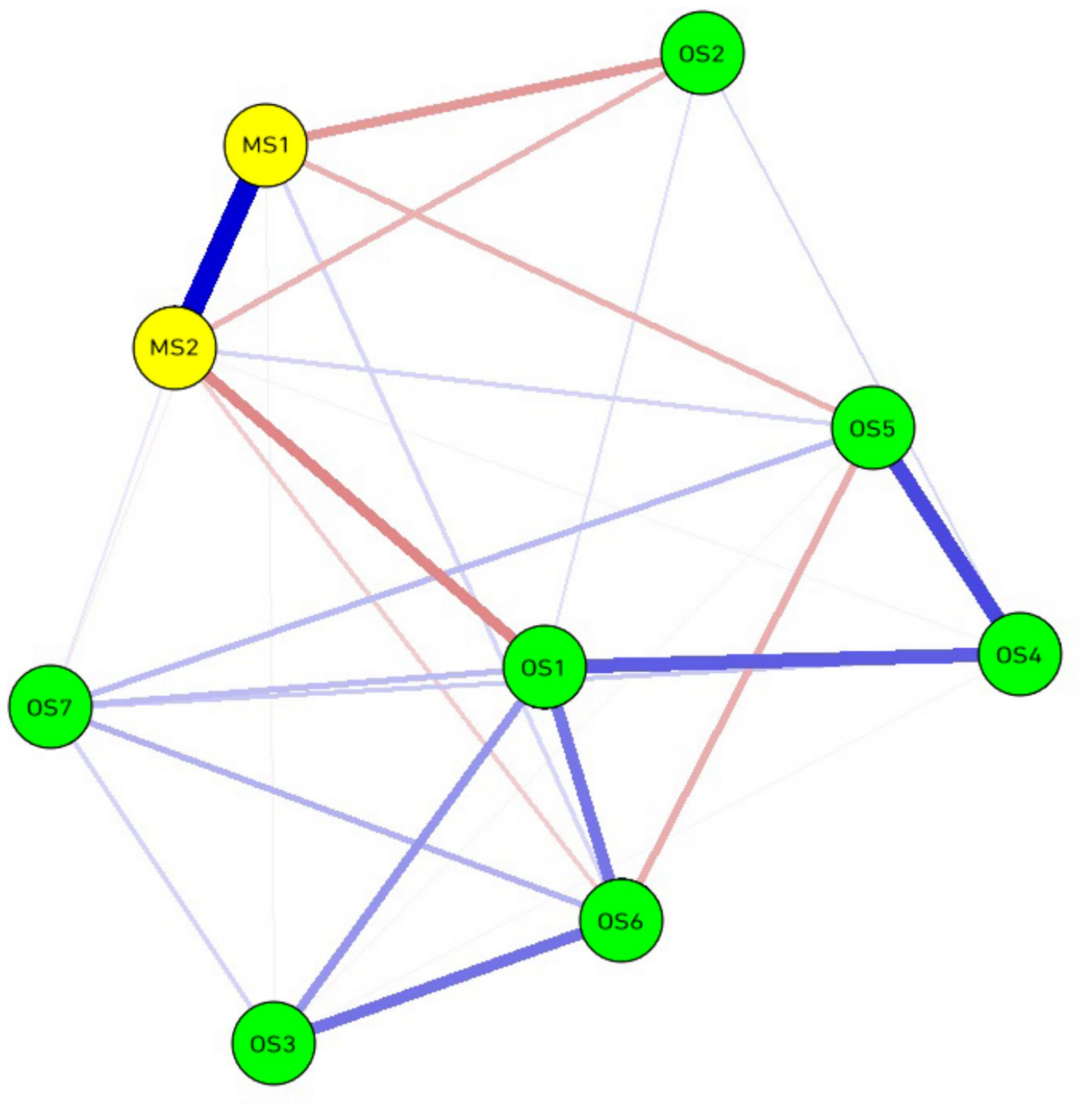
Estimated partial correlation networks of work stressors and job satisfaction.

In the visualizations of the estimated partial correlation networks shown in Figure 1, nodes representative of occupational stress and job satisfaction exhibit densely interconnected patterns. Approximately 22% of all network edges were nullified. Specifically, within the entire network, organizational management (MS1) – occupational interests (MS2) displays the strongest association with a marginal weight of 0.588. Within the occupational stress group, the strongest connection is observed between career development (OS4) and interpersonal relationships (OS5), with a marginal weight of 0.471, closely followed by the relationship between career development (OS4) and intrinsic satisfaction (OS1) at a marginal weight of 0.371. Additionally, in terms of negative correlations, the most pronounced connection is between extrinsic satisfaction (MS2) and intrinsic satisfaction (OS1), with a marginal weight of −0.274, while the next significant negative correlation is between organizational management (MS1) and occupational interests (OS2) with a marginal weight of −0.233.

**Figure 2:**
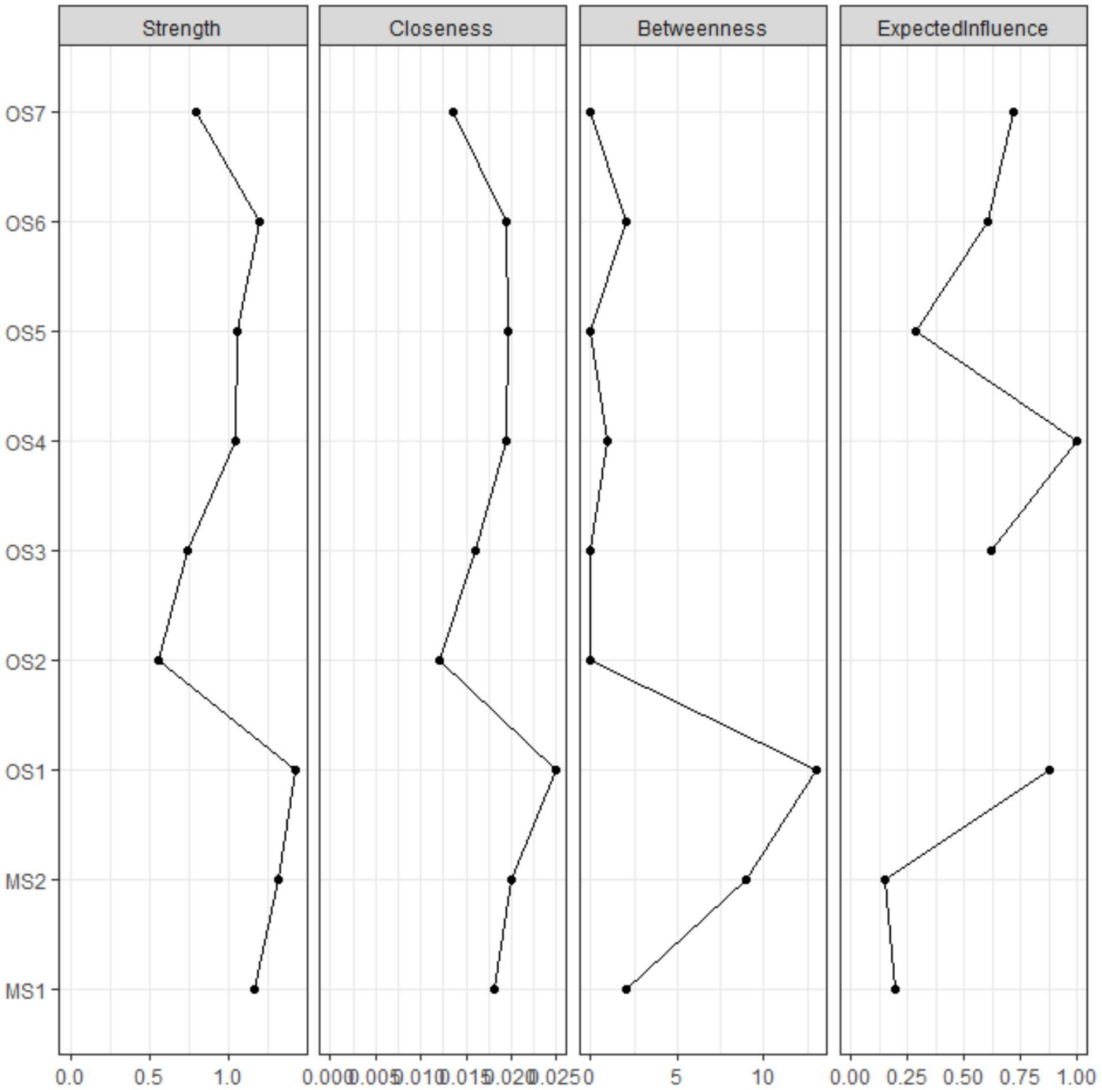
Centrality indices of node strength, closeness and betweenness, expected influence of the estimated network. All indices are shown as standardized z-scores.

To assess the centrality of individual nodes within the integrated network, metrics such as node strength, node closeness, and node betweenness were calculated. Figures depicting these three standardized centrality indices for the amalgamated partial correlation network can be found in Figure 2. Intrinsic satisfaction (OS1) emerges as a crucial was the most central symptom across all centrality indices, followed by the extrinsic satisfaction (MS2).

**Figure 3:**
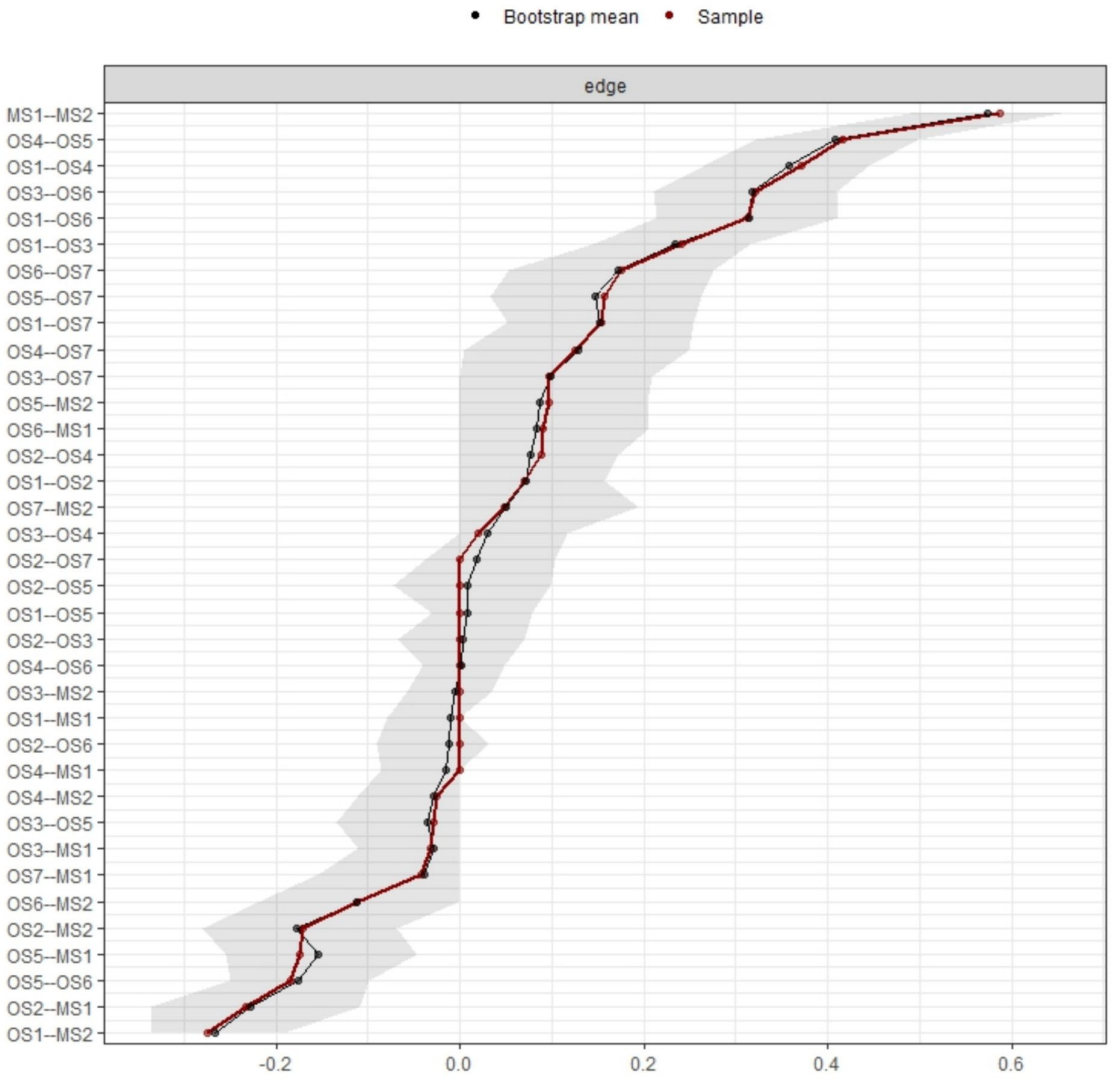
Bootstrapped confidence intervals of estimated edge-weights (top panel).

**Figure 4:**
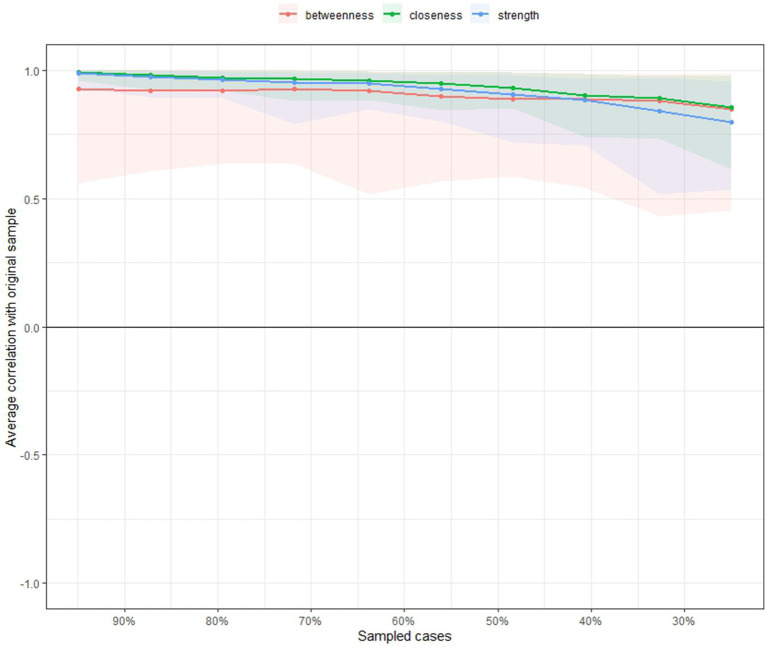
Stability of the network model.

An edge weight bootstrap procedure was conducted on the 95% confidence intervals surrounding the edge weights to evaluate the accuracy of the combined estimated network. Figures 3, 4 illustrates the visualization of the edge weight bootstrap procedure for assessing network accuracy. The results indicate relatively small bootstrapped confidence intervals around most estimated edge weights, suggesting high accuracy. Larger bootstrapped confidence intervals imply that caution should be exercised when interpreting the order of these edges in the network. To assess the stability of the combined estimated network, a case-dropping subset bootstrap procedure was conducted to derive the network model based on data subsets. A visualization of the case-dropping bootstrap process for bridge strength and expected bridge influence is depicted in Figure 4. Quantifying network stability through CS coefficients, findings from the case-dropping bootstrap suggest that node strength stands out as the most consistent centrality metric [CS(cor = 0.7) = 0.593].

There were no significant differences found between the LASSO networks for male and female participants in terms of global strength (Test statistic S: 0.655; *p* = 0.209) and structural invariance (Test statistic M: 0.219; *p* = 0.364) based on Network Comparison Test permutation tests.

## Discussion

This study presents a pioneering analysis of occupational stress and job satisfaction symptom networks within a sample of radiologists. The findings indicate variations in the strength of associations among different symptoms, highlighting that certain symptoms may have more significant connections than others. Additionally, individual factors contributing to occupational stress and job satisfaction are not uniformly crucial within the network. Notably, connections between symptoms within each domain were generally stronger than those between different domains. The stability of both network edges and the strength centrality metric lends confidence to the conclusions drawn from these cross-sectional networks. Overall, this study contributes valuable insights into the intricate relationships among occupational stress and job satisfaction symptoms among radiologists, shedding light on potential areas for intervention and support strategies.

It’s noteworthy that the strongest positive correlation is observed between organizational management and occupational interests, with a marginal weight of 0.588. This finding suggests that effective organizational management is closely intertwined with individual occupational interests in the work environment of radiologists. When doctors perceive the management of their organization to be efficient and supportive (30), they are more likely to find alignment between their daily work and personal interests, thereby enhancing job satisfaction and engagement (31, 32). Within the occupational stress group, another prominent finding is the strong connection between career development and interpersonal relationships (marginal weight of 0.471). This indicates that radiologists place a high value on maintaining positive relationships with colleagues and superiors while pursuing career advancement. Opportunities for career development and smooth interpersonal relationships may work together to alleviate doctors’ occupational stress and increase their job satisfaction. Therefore, organizations should strive to create a work environment that fosters both personal growth and team collaboration.

The exploration of centrality indices within the integrated network yields valuable insights into the key symptoms impacting both occupational stress and job satisfaction among radiologists. In particular, intrinsic satisfaction (OS1) emerges as a crucial node across all three centrality metrics, highlighting its foundational significance in shaping the work experience of radiologists. High levels of intrinsic satisfaction are linked to greater fulfillment and engagement at work, which can positively affect overall job satisfaction and help alleviate occupational stress (33, 34). The strong centrality of intrinsic satisfaction suggests that improving this aspect of work life could have far-reaching impacts on job satisfaction and well-being among radiologists. Similarly, extrinsic satisfaction (MS2) holds a prominent position in the network, closely following intrinsic satisfaction in terms of centrality. While extrinsic factors like salary and benefits are crucial drivers of job satisfaction, their impact may be secondary to the intrinsic rewards of the work itself (35). Nonetheless, the significant centrality of extrinsic satisfaction underscores its importance in the overall job satisfaction landscape, particularly concerning the core needs and expectations of radiologists in their professional domains.

This study faces several noteworthy limitations. Firstly, its cross-sectional design constrains result extrapolation and the ability to infer causality. Further longitudinal research is vital for a deeper understanding of the intricate relationship between occupational stress and job satisfaction symptoms. Secondly, participant recruitment via convenient sampling from a single location in China potentially limits the generalizability of findings to a broader, nationally representative sample. Thirdly, inherent reporting and recall biases may persist, underscoring the necessity for caution in result interpretation. In this study, we acknowledge a significant limitation, which is the absence of an international perspective on the same study cohort. Due to the scope of the research and resource constraints, we were unable to explore the international aspect in depth. In future research, we aim to broaden our perspective, including more international participation, to offer a more comprehensive and diverse analysis.

## Conclusion

In conclusion, this study sheds light on the intricate relationships between occupational stress and job satisfaction among radiologists, emphasizing the importance of effective organizational management, interpersonal relationships, and opportunities for career development in mitigating stress and enhancing job satisfaction. Addressing the identified limitations through longitudinal research and diverse sampling methods is crucial for advancing our understanding in this area and for developing tailored interventions to support radiologists’ well-being and professional fulfillment.

## Data availability statement

The original contributions presented in the study are included in the article/supplementary material, further inquiries can be directed to the corresponding author.

## Ethics statement

The studies involving humans were approved by Approval for this study was obtained from the Ethics Committee of Nantong First People's Hospital with the identification number 2022KT262. The participants provided their informed consent. The studies were conducted in accordance with the local legislation and institutional requirements. The participants provided their written informed consent to participate in this study.

## Author contributions

JJ: Writing – original draft, Writing – review & editing. BH: Writing – original draft, Writing – review & editing, Funding acquisition. SG: Writing – original draft. MS: Writing – original draft. XR: Writing – original draft.
